# Role of Natural Killer and Gamma-Delta T cells in West Nile Virus Infection

**DOI:** 10.3390/v5092298

**Published:** 2013-09-20

**Authors:** Tian Wang, Thomas Welte

**Affiliations:** 1 Department of Microbiology & Immunology, University of Texas Medical Branch, Galveston, TX 77555, USA; E-mail: thomaswelte2007@gmail.com; 2 Department of Pathology, Sealy Center for Vaccine Development, Center for Biodefense and Emerging Infectious Diseases, and Institute for Human Infections and Immunity, University of Texas Medical Branch, Galveston, TX 77555, USA

**Keywords:** West Nile virus, Natural Killer cells, Gamma-delta T cells

## Abstract

Natural Killer (NK) cells and Gamma-delta T cells are both innate lymphocytes that respond rapidly and non-specifically to viral infection and other pathogens. They are also known to form a unique link between innate and adaptive immunity. Although they have similar immune features and effector functions, accumulating evidence in mice and humans suggest these two cell types have distinct roles in the control of infection by West Nile virus (WNV), a re-emerging pathogen that has caused fatal encephalitis in North America over the past decade. This review will discuss recent studies on these two cell types in protective immunity and viral pathogenesis during WNV infection.

## 1. Introduction

West Nile virus (WNV), a plus-sense, single-stranded neurotropic flavivirus, has been a public health concern in North America for more than a decade [1,2]. The virus is maintained in an enzootic cycle that involves mosquitoes and birds, with humans and horses as incidental hosts. Infection in humans results from mosquito bites, blood transfusion, organ transplantation, breast feeding, and *in utero* or occupational exposure [2,3,4,5,6]. WNV infection of the central nervous system (CNS, neuroinvasive disease) commonly presents as encephalitis, meningitis, or acute flaccid paralysis. The overall mortality rate in persons who develop WNV neuroinvasive disease is about 10%, although the mortality rate increases significantly in the elderly and immunocompromised. Recently, some WNV convalescent patients were reported to have significant long-term morbidity years after their acute illness; symptoms include muscle weakness and pain, fatigue, memory loss, and ataxia [7,8,9,10,11]. At present, there is no specific therapeutic agent for treatment of the infection. No approved human vaccines are available for its prevention.

WNV has been studied in various animal models, including mice, hamsters, monkeys, and horses [12,13,14,15]. The murine model is an effective *in vivo* experimental model to investigate viral pathogenesis and host immunity in humans. Following the initial subcutaneous or intraperitoneal inoculation in mice, WNV induces a systemic infection and eventually invades the CNS. Mice die rapidly when encephalitis develops, usually within one to two weeks. The severity and symptoms of lethal infection observed in the murine model mimic the symptoms caused by WNV infection in humans [13,16,17]. Studies from experimental animal models, *in vitro* cell culture, and/or WNV patient samples have provided important insights into host immunity to WNV infection. Natural killer (NK) cells and γδ T cells are two innate lymphocytes that respond rapidly and non-specifically to viral infection. They are also known to form a unique link between innate and adaptive immunity. Moreover, the characteristics of these two cell types in adaptive immunity have been described in several disease models [18,19,20,21]. In this review, we will discuss recent studies on these two unique cell types in both protective immunity and viral pathogenesis during WNV infection.

## 2. NK Cells

NK cells are important for early immune reactions against viral infections and cancer. They are a subset of lymphocytes that provide innate effector mechanisms through secretion of cytokines and direct cytotoxic effects, which are triggered by releasing cytotoxic granules containing perforin and granzymes [22].

### 2.1. NK Cells in Host Immunity to WNV Infection

NK cells have been reported to be involved in the host immunity to infection of many flaviviruses, including yellow fever virus, Japanese encephalitis virus, tick-borne encephalitis virus, dengue virus, and WNV [23,24,25,26]. Vargin *et al*. [25] were the first to report that infection of mice with WNV was accompanied by the temporary activation of NK cells in the spleen. Similar to other innate immune cells, such as monocytes and dendritic cells (DCs), NK cells can migrate into the CNS as the virus invades the brain, following a systemic infection in mice [27]. They presumably play an important role in the control of WNV infection by their recognition and elimination of infected cells, as demonstrated in *ex vivo* cytotoxicity assays reported by several groups. In one study [28], brain leukocytes isolated from WNV-infected mice displayed an NK cell phenotype and had the ability to lyse WNV-infected and non-infected cell lines *in vitro*. Another group showed that active human NK cells, which resulted from co-cultivation of peripheral blood mononuclear cells (PMBCs) with radiation-killed K562-mb15-41BBL stimulatory cells inhibited WNV infection in Vero cells [29].

There are also conflicting reports about the functional significance of NK cells in WNV-infected hosts. For example, WNV-infected mouse astrocytes were shown to be resistant to NK cell lysis, which was indicative of a low susceptibility of CNS cells to NK cells during WNV infection [30]. When NK cells were depleted in mice by using an antibody against NK1.1, the mice neither became more susceptible to WNV nor was there an increased morbidity. Furthermore, the Ly49a transgenic strain of mice, which is deficient in circulating NK cells, also showed a susceptibility to WNV similar to that of wild-type mice [31]. Thus, the role of NK cells during *in vivo* WNV infection remains controversial. There are a few possible reasons to explain this complexity. First, the divergent phenotypic and functional features of NK cells are often influenced by organ-specific factors, including local microenvironment and unique cellular interactions [32]. This has been well documented in several disease models. During coronavirus infection or in autoimmune disease, CNS-specific NK cells provided protection against encephalitis, either by reducing viral replication or inhibiting the activation of autoimmune T cells through killing of activated microglia [33,34]; whereas during chronic hepatitis C virus infection, hepatic NK cells were found to inhibit liver fibrosis and tissue regeneration by promoting stellate cell death [35]. Likewise, the CNS-specific NK cells may have distinct functions during *in vivo* infection other than direct killing of WNV-infected local cells. Interestingly, a recent study [36] showed that NK cells were capable of preventing the spread of WNV infection only to certain mouse tissues, such as the liver, but not the spleen. Additionally, WNV could develop strategies in evasion of NK-cell mediated killing during *in vivo* infection. NK cell activation is regulated by the balance of activating and inhibitory receptors on its surface. The inhibitory receptors killer cell immunoglobulin-like (KIR) receptors in humans, the lectin-like Ly49 (mouse), and the CD94-NKG2A dimers bind to major histocompatibility complex (MHC) class I molecules. Infection of mouse or human cells with flaviviruses is known to increase the cell-surface expression of MHC class I [37,38]. In particular, WNV infection upregulates MHC class I expression by enhancing the transport activity of TAP and by NF-κB- dependent transcription activation of MHC class I genes [39,40,41]. Therefore, WNV may evade NK-cell mediated killing by upregulation of MHC class I on infected cells. Lastly, although the depletion approach has been commonly used to determine the functional role of NK cells during *in vivo* flavivirus infection [31,42], it has been reported that NK cells can’t be fully depleted in all WNV- infected tissues [36].

### 2.2. Factors that Contribute to NK Cell-Activation and Killing during WNV Infection

Activated NK cells inhibited WNV infection of Vero cells through both antibody-dependent cellular cytotoxicity (ADCC) and non-cytolytic activities, such as secretion of interferon (IFN)-γ [29]. Both host factors and microbial signals are involved in triggering NK cell activation during viral infection. NK cells can detect microbial signals by stimulation of their pathogen recognition receptors (PRRs), such as toll-like receptor (TLR) or RIG-I like receptor (RLR), or being activated by innate cytokines predominantly produced by infected DCs [43,44]. By using the integrated system biology approach, Suthar *et al*. [36] have recently demonstrated that the liver-specific NK cell activation during WNV infection is regulated by two innate immune signaling pathways, including RLR and IFN. In WNV-infected liver, IFN functions intrinsically in regulation of NK cell activation, proliferation, and maturation; whereas RLR-mediated type 1 IFN production in DCs imparts signaling extrinsically via crosstalk to NK cells and triggers their activation. NK cells are also known to sense microbial and non-microbial signals from target cells through a variety of activating and inhibitory receptors, which influence cytotoxicity towards virus-infected cells and cancerous cells [45]. Upon WNV infection, interaction of the activating receptor NKp44 of human NK cells with domain III of the WNV envelope (E) protein was shown to be an important step in triggering both IFN-γ secretion and cytolytic activity of NK cells during infection [46]. 

## 3. γδ T Cells

γδ T cells comprise a minority of the CD3^+^ T cells in lymphoid tissue and blood, but are well represented at epithelial and mucosal sites [47]. Unlike αβ T cells, they lack MHC restriction and have the potential capacity to respond to antigens without a requirement for conventional antigen processing [48]. In response to microbial antigens, γδ T cells can rapidly produce cytokines, such as IFN-γ, tumor necrosis factor (TNF)-α and interleukin (IL)-17 [49,50]. γδ T cells are divisible into functionally distinct subsets, which distribute in an organ-specific manner. In mice, γδ T cells include mostly Vγ1, Vγ2, Vγ4, Vγ5, Vγ6, and Vγ7 subsets [51], which have direct and indirect effects on host immunity to many infectious pathogens [52,53].

### 3.1. The Protective Effects of γδ Τ Cells in Innate and Adaptive Immunity against WNV

γδ T cells are important for protective immunity against WNV infection. TCRδ^−/−^ mice, which are deficient in γδ T cells, had elevated viremia, as well as more severe encephalitis, and were much more susceptible to WNV infection than were the wild-type controls [54]. The IFN-γ producing activity partially contributes to their protective effect in host immunity (Table 1). Upon WNV infection, γδ T cells quickly expanded as early as day two and are the major resource for producing IFN-γ. Furthermore, transfer of splenocytes from TCRβ^−/−^ IFNγ^−/−^ mice, which have a defect in the IFN-γ-producing capacity of γδ T cells, did not affect host susceptibility in TCRδ^−/−^ mice [54]. Another group also demonstrated that irradiated mice reconstituted with IFN-γ-deficient γδ T cells had significantly higher levels of viral loads in the blood and brains during WNV infection than mice reconstituted with IFN-γ-sufficient γδ T cells [55]. Vγ1^+^ cells were the major γδ subset producing IFN-γ. Mice depleted of Vγ1^+^ cells displayed a phenotype similar to that observed in TCRδ^−/−^ mice in response to WNV infection ([56], Table 1). Cytolytic function is another important mechanism of viral control attributed to γδ T cells [57,58,59]. TCRδ^−/−^ mice had reduced levels of intracellular perforin in splenocytes at day six, post-WNV infection, implying their contribution to cytolytic activity (Table 1) [54]. Both CD4^+^ and CD8^+^ T cells have been shown to be responsible for the cytolytic activity detected in splenocytes during the first week of WNV infection [60,61]. We have noted that splenic CD4^+^ T cells of TCRδ^−/−^ mice had a lower cytotoxicity potential than those of wild-type mice [62]. Thus, γδ T cells may contribute to the cytolytic activity against WNV in the periphery directly and/or indirectly by regulation of αβ T cell response. 

γδ T cells also play a role in memory T cell development during WNV infection. TCRδ^−/−^ mice that survived primary WNV infection had a numeric and functional reduction in memory T cell responses. However, γδ T cells are not directly involved in the recall response to WNV infection. This is supported by the fact that depletion of these cells in WNV-infected mice does not affect host susceptibility to the secondary challenge [63]. In WNV-infected ΤCRδ^−^^/−^ mice, splenic DCs had an impaired antigen-presenting capacity, and lower levels of CD40, CD80, CD86, and MHC class II expression and IL-12 production than did those of wild-type mice [64]. This suggests that the crosstalk between γδ T cells and DCs plays an important role in promoting DC maturation and T cell priming. A low level of WNV replication in γδ T cells induced pro-inflammatory cytokines, including IFN-γ, and TNF-α (Table 1). DCs co-cultured with WNV-infected γδ T cells also had enhanced levels of co-stimulatory molecules, MHC class II expression, and IL-12 production [64]. Thus, WNV-activated γδ T cells promote DC activation through direct contact with DCs and/or the secreting molecules induced upon infection.

**Table 1:** γδ T cells in host immunity to West Nile virus (WNV) infection.

Functionality	Role in host immunity	Major αβ subsets involved
CTL activity	Control of WNV dissemination	? detected within the first week
Production of IFN-γ	Control of WNV dissemination; Promote DC maturation	Vγ1^+^ cells, as early as day 2
Production of TNF-α	Promote DC maturation and activation; Contribute to BBB compromise and increase viral load in the CNS	Vγ4^+^ cells
Production of IL-17	Not known	Vγ4^+^ cells
Production of IL-10	Increase viral infection and mortality during WNV infection	Mostly by CD4^+^αβ T cells and suppressed by Vγ1^+^ cells
Production of TGF-β	Suppress Vγ1^+^ cell expansion in the periphery and their infiltration into the CNS	Vγ4^+^ cells
Anti-inflammation	Reduce inflammation in the CNS	Vγ1^+^ cells

### 3.2. γδ T Cells in WNV Pathogenesis

A major risk factor for fatality due to WNV infection in humans is aging [65,66]. Aged mice were more susceptible to WNV infection than young adult mice [56,67]. Vγ1^+^ cells of aged mice displayed a slower, reduced response to WNV infection compared to those of young adult mice, suggesting dysfunction of γδ T cells contribute to host susceptibility to WNV encephalitis [56]. Immunotherapy can improve the anti-tumor effect of γδ T cells [68,69]. In one study [70], oral administration of active hexose correlated compound (AHCC), an extract of *Lentinula edodes* of the *Basidiomycete* family of fungi rich in α-glucans, enhanced the protective Vγ1^+^ T cell response and thus attenuated viremia in aged mice following lethal WNV infection.

Aged mice, when compared to young adult mice, also displayed a higher content of Vγ4^+^ cells, another major subpopulation of peripheral γδ T cells. Depletion of Vγ4^+^ cells in young mice resulted in a lower mortality following WNV-induced encephalitis [56]. The pathogenic effects of Vγ4^+^ cells are mediated via the production of proinflammatory and regulatory cytokines during WNV infection ([56,71], see Table 1). TNF-α has been reported to be responsible for BBB compromise and WNV entry into the brain [72]. Vγ4^+^ cell-depleted mice had reduced TNF-α levels in the CNS, accompanied by a decreased viral load in the brain and a lower mortality to WNV encephalitis [56]. Vγ4^+^ cells also produced IL-17, which is known to increase inflammation by recruiting cells, such as neutrophils or macrophages, to infection sites in several disease models [73,74]. Nevertheless, the IL-17- producing activity is considered to be dispensable for host immunity against WNV, as *in vivo* blocking of IL-17 signaling did not affect host susceptibility to WNV infection [71]. Vγ4^+^ cells also negatively regulate Vγ1^+^ T cell responses during WNV infection via the production of TGF-β (Table 1). This effect contributes directly to higher viremia, which leads to more virus dissemination into the CNS, and induction of encephalitis [56,71]. Additionally, the suppressive effects of Vγ4^+^ cells are associated with decreased IL-10 levels and reduced inflammation in the CNS ([71], Table 1). IL-10 plays a pathogenic role in host immunity to WNV infection [75,76]. Vγ1^+^ cells have been shown to suppress the IL-10-producing CD4^+^CD25^+^ T cells in the lungs of ovalbumin-sensitized and challenged mice [77]. Moreover, Vγ1^+^ T cells are known to decrease inflammation during bacterial and coxsackievirus infection [52,78].

### 3.3. Factors that Contribute to γδ T Cell Activation during WNV Infection

There are few antigens reported to be recognized by γδ T cell receptors [79]. TLRs sense different pathogen–associated molecular patterns (PAMP) during microbial infection [80,81]. The expression of PRRs, such as TLR2, TLR3, TLR4, and TLR7/8, on γδ T cells has been reported [82,83,84,85]. Among them, TLR3- and TLR7-induced innate cytokine responses are involved in both protection and pathogenesis during WNV infection [72,86,87,88]. Myeloid differentiation factor 88 (MyD88), the primary adaptor for most TLRs, also restricts WNV by inhibiting replication in subsets of cells and modulating immune cell migration into the CNS [89]. We have recently found that γδ T cells of MyD88 and TLR -deficient mice, had a reduced expansion and activation compared to those of wild-type mice following WNV infection, which indicates a role of MyD88-dependent PRR signaling in γδ T cell activation [90]. TLRs are known to directly or indirectly contribute to γδ T cell activation. TLR ligands could act as co-stimulatory signals for TCR-activated human γδ T cells [91,92]. Alternatively, γδ T cells and DCs also exert regulatory influences on each other. For example, induction of human γδ T cells by poly I:C, a ligand for TLR3, depends on DCs mediated by Type-1 IFNs [93]. Human γδ T cells were activated by plasmacytoid DCs upon infection by another important flavivivirus, yellow fever virus [94]. Whether γδ T cells are induced by WNV directly via their innate immune receptors, such as TLRs, or indirectly by interaction with TLR-expressing innate immune cells remains under investigation. 

## 4. Conclusions

In summary, NK cells and γδ T cells play both similar and dissimilar roles in host immunity to WNV infection (Table 2). They both respond rapidly and non-specifically to WNV infection and control viral dissemination by secretion of IFN-γ and/or cytolytic activity. Both cell types cross-talk with DCs during WNV infection, which results in DC maturation and NK cell activation [36,64]. PRR-mediated innate immune signaling pathways, including those used by TLR, RLR-, or IFN, are important for regulation of NK cell and γδ T cell activation during WNV infection. Furthermore, γδ T cells regulate memory T cell development by promoting DC maturation and activation. The role of NK cells in adaptive immunity to WNV infection remained undefined. γδ T cell subsets display distinct functions in both protection and pathogenesis upon WNV infection. The role of NK cells during *in vivo* WNV infection is complex and sometimes paradoxical. The functional significance of NK cells in WNV susceptibility should be investigated in an organ-specific manner, with a full characterization of multiple activating and inhibitory receptors on the NK cell surface. Due to their unique role in host innate and adaptive immunity, studies of the functionality of NK cells and γδ T cells and their cross-talk with DCs will provide important insights into WNV immunotherapy and vaccine development for the potential target population. 

**Table 2:** Comparison of Natural Killer (NK cells) and γδ T cells in host immunity to WNV infection.

Category	NK cells	γδ T cells
CTL	Direct cytotoxicity, ADCC and IFN-γ production	Direct cytotoxicity, and IFN-γ production
Cross-talk with DCs	Yes, this triggers NK cell activation	Yes, this leads to DC maturation
Role in Adaptive Immunity	Not known	Regulate T cell response via promoting DC maturation and activation
Subsets Diversity	Not known	Vγ1 and Vγ4 subsets have distinct roles in protection and pathogenesis
Organ-specific function	Yes, mouse liver and spleen NK cells have different roles during WNV infection	Not known
Innate immune signaling pathways involved	RIG-I, IFN	MyD88-dependent PRRs
Host studied	Human, mouse	Mouse
